# Genetic Diversity, Community Assembly, and Shaping Factors of Benthic Microbial Eukaryotes in Dongshan Bay, Southeast China

**DOI:** 10.3389/fmicb.2020.592489

**Published:** 2020-12-23

**Authors:** Rong Gu, Ping Sun, Ying Wang, Fengling Yu, Nianzhi Jiao, Dapeng Xu

**Affiliations:** ^1^ State Key Laboratory of Marine Environmental Science, Xiamen University, Xiamen, China; ^2^ College of Ocean and Earth Sciences, Xiamen University, Xiamen, China; ^3^ Fujian Key Laboratory of Marine Carbon Sequestration, Xiamen University, Xiamen, China; ^4^ Key Laboratory of the Ministry of Education for Coastal and Wetland Ecosystem, College of the Environment and Ecology, Xiamen University, Xiamen, China; ^5^ Fujian Provincial Key Laboratory for Coastal Ecology and Environmental Studies, Xiamen University, Xiamen, China

**Keywords:** dispersal limitation, high throughput sequencing, protist, spatial heterogeneity, SSU rRNA gene, co-occurrence network

## Abstract

Microbial eukaryotes are pivotal components of marine ecosystems. However, compared with the pelagic environments, the diversity distribution and the driving mechanisms of microbial eukaryotes in the marine sediments have rarely been explored. In this study, sediment cores were collected along a transect from inner to outer Dongshan Bay, Southeast China. By combining high throughput sequencing of small-subunit (SSU) rRNA gene with measurements on multiple environmental variables, the genetic diversity, community structure and assembly processes, and environmental shaping factors were investigated. Alveolata (mainly Ciliophora and Dinophyceae), Rhizaria (mainly Cercozoa), and Stramenopiles (mainly Bacillariophyta) were the most dominant groups in terms of both relative sequence abundance and operational taxonomic unit (OTU) richness. Grain size composition of the sediment was the primary factor determining the alpha diversity of microbial eukaryotes followed by sediment depth and heavy metal, including chromium (Cr), zinc (Zn), and plumbum (Pb). Geographic distance and water depth surpassed other environmental factors to be the primary factors shaping the microbial eukaryotic communities. Dispersal limitation was the primary driver of the microbial eukaryotic communities, followed by drift and homogeneous selection. Overall, our study shed new light on the spatial distribution patterns and controlling factors of benthic microbial eukaryotes in a subtropical bay which is subjected to increasing anthropogenic pressure.

## Introduction

Microbial eukaryotes (protist) are critical components of the microbial food webs and play pivotal roles as primary producers, consumers, decomposers, and trophic links in influencing the biogeochemical processes in the marine aquatic systems (Worden et al., 2015; Caron et al., 2016). They are also pivotal components of the marine sediment ecosystems and play diverse roles in maintaining ecosystem function and biogeochemical cycling (Orsi, 2018). For example, in tidal flats, diatoms form a major component of microphytobenthos on sediments and are major players in nutrient cycling and provide a food resource to larger grazers (Lohrer et al., 2004; Evrard et al., 2012). Generally, the abundances of sediment bacteria are several orders of magnitude higher than those in the pelagic environments (Rublee, 1982), so do the bacterial productions (Starink et al., 1996), and grazing of heterotrophic microbial eukaryotes (e.g., heterotrophic nanoflagellates and ciliates) on bacteria was proposed to be the primary cause of bacterial mortality (Fenchel, 1978; Pasulka et al., 2017). In addition, the abundances of some groups of microbial eukaryotes, e.g., nanoflagellates and ciliates, may even exceed those of the overlaying sea water (Wickham et al., 2000; Meng et al., 2011).

Compared with the pelagic environments, the diversity of microbial eukaryotes in the marine sediments has rarely been explored which is probably due to the methodological difficulties including but not restricted in the sample collection, isolation of species, extraction of nucleic acids, specific techniques and equipment needed, and taxonomic expertise if microscopy-based approaches are applied. In the last several decades, the development of the culture-independent high throughput sequencing (HTS) approaches offered a technical alternative to solve, at least partially, the above problems (Hudson, 2008). HTS has expanded our knowledge of the genetic diversity and community composition of microbial eukaryotes in sediments of coastal oceans (Gong et al., 2015; Massana et al., 2015; Salonen et al., 2019), hydrothermal vents (Edgcomb et al., 2002; López-García et al., 2007; Brisbin et al., 2019; Murdock and Juniper, 2019), cold seep (Sauvadet et al., 2010; Pasulka et al., 2016), deep-sea (Bik et al., 2012; Wu and Huang, 2019), and anoxic environments (Dawson and Pace, 2002; Stoeck and Epstein, 2003; Takishita et al., 2005). Based on the limited amount of data collected so far, it has been proposed that marine sediments harbor huge, yet largely unknown diversity of microbial eukaryotic assemblages whose diversity may be comparable or even exceed that of the planktonic groups (Scheckenbach et al., 2010; Bik et al., 2012; Forster et al., 2016). The diversity of benthic microbial eukaryotes was thus considered “the under-charted majority” (Forster et al., 2016).

Compared with the deep-sea floor, physicochemical factors, such as light, temperature, and salinity, usually change dramatically in coastal oceans over short periods. The complex and changeable environment in coastal waters may greatly impact the diversity, distribution, and community composition of benthic microbial eukaryotes. For example, anoxic sediments are hostile environments for many eukaryotes because of low oxygen concentrations and high concentrations of toxic substances, including hydrogen sulfide (H_2_S; Bagarinao, 1992). In the meantime, some coastal areas are subjected to increasing anthropogenic pressure, including the discharge of pollutants, e.g., heavy metals, nutrients, etc. Thus, investigating the spatial distribution patterns and community assembly processes, and environmental driving factors can help us better understand the responses of benthic microbial eukaryotes to changing environments.

In this study, Dongshan Bay, which is located in Southeast China and has undergone the influence of increasing anthropogenic activities such as the discharge of domestic and aquacultural wastewater in offshore areas (Ni et al., 2015), was chosen to investigate the impact of environmental variables on benthic microbial eukaryotic communities. A metabarcoding approach by applying HTS on the V4 hypervariable regions of the small-subunit (SSU) rRNA gene was employed to study the benthic microbial eukaryote communities along a transect from inner to outer bay and at each sampling sites, sediments were collected at 1 cm depth interval down to 5 cm depth. By measurements on multiple environmental variables, the main aims of our study were: (i) to explore the genetic diversity, community composition, and assembly processes of benthic microbial eukaryotic community across spatial scales and (ii) to infer the environmental factors shaping the alpha- and beta-diversities of benthic microbial eukaryotic communities.

## Materials and Methods

### Sample Collection

**Figure 1:**
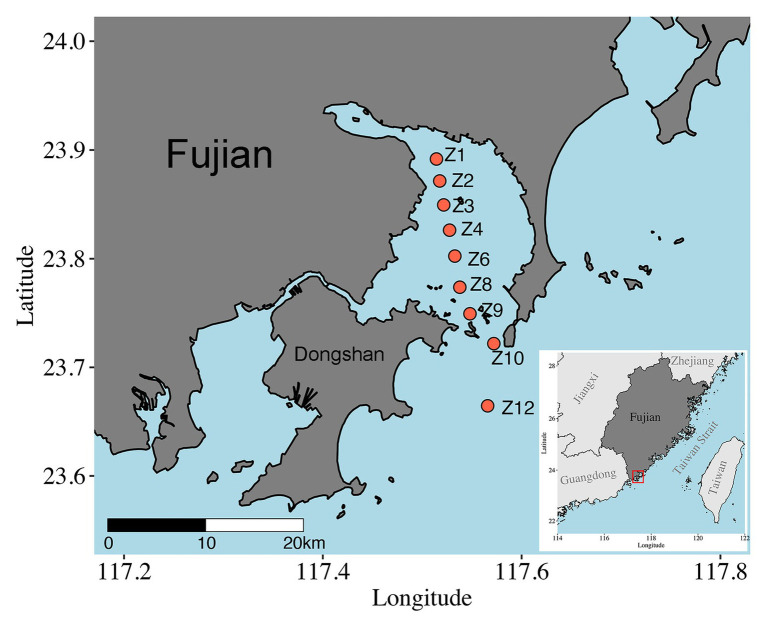
Map of sampling sites in Dongshan Bay, Fujian Province, China.

Samples were collected onboard *R/V Ocean II* along a transect across Dongshan Bay, Southeast China, on May 28, 2018 (Figure 1). Nine sites were visited, and sediments were collected using a Hydro-Bios Lenz bottom sampler (Bottom Sampler acc. to Lenz, Germany). After slowly and carefully retrieved on board, sediment was collected with a custom-made PVC corer, and the upper 5 cm with 1 cm intervals were subsampled, immediately frozen with liquid nitrogen, and stored at −80°C until DNA extraction. At Z10 and Z12, only surface (1 cm) samples were obtained. A total of 37 samples were retrieved (Supplementary Table S1). The depth, temperature, and salinity of bottom water were measured *in situ* using CTD sensors (SeaBird SBE 911plus, Sea-Bird Electronics, WA, United States).

### Metal Analysis

Concentrations of metal, including arsenic (As), cadmium (Cd), chromium (Cr), copper (Cu), hydrargyrum (Hg), plumbum (Pb), and zinc (Zn), were determined according to Buccolieri et al. (2006). In brief, ca. 0.5 g of sediment was microwave-digested in a mixture of nitric acid (9 ml) and hydrochloric acid (3 ml) at ca. 170°C for 1 h. After digestion and cooling, samples were diluted with boric acid, filtered, and analyzed by an inductively coupled plasma atomic emission spectrometer (ICP-AES; Buccolieri et al., 2006).

### Grain Size Analysis

For grain size analysis, surface (1–5 cm depth) sediment samples were pretreated with H_2_O_2_ (around 20 ml, 20%) for organic removal and then with 1 mol/L HCl (around 10 ml) for carbonate removal. After the reaction completed, the supernatant was removed, and the 5% (NaPO_3_)_6_ (about 20 ml) were added and stay for 24 h for a complete dispersal of the grains. The pretreated samples were then measured for grain size composition using the Malvern Mastersizer 3000 laser diffraction particle size analyzer. The relative error of duplicated measurements is ≤1.0%. Based on the results of grain size analysis, sediments were classified and named according to the classification scheme suggested by Shepard (1954): Clay is the fraction with grain size <4 μm; silt is the fraction with grain size 4–63 μm; and sand is the fraction with grain size >63 μm.

### DNA Extraction, PCR Amplification, and High Throughput Sequencing

Total DNA from each sample (approximately 0.25 ~ 0.5 g) was extracted using the DNeasy PowerSoil Kit (Qiagen, Germany) according to the manufacturer’s protocol. The quality and concentrations of the extracted DNA were determined using a Nanodrop 2000c spectrophotometer (ThermoFisher, United States). The V4 hypervariable region of the SSU rRNA gene (ca. 370 bp) was amplified using primers TAReuk454FWD1 (5'-CCA GCA (G/C)C(C/T) GCG GTA ATT CC-3') and TAReukREV3 (5'-ACT TTC GTT CTT GAT (C/T)(A/G)A-3'; Stoeck et al., 2009). PCR amplicons were purified using Wizard® SV Gel and PCR Clean-Up System (Promega, Beijing, China). Paired-end sequencing (2 × 250 bp) was performed at Majorbio (Shanghai, China) using an Illumina MiSeq platform. Sequences obtained have been submitted to the NCBI Sequence Read Archive and are available through the accession number PRJNA645757.

### Sequence Processing

Sequences were processed and analyzed using QIIME v.1.8.0 (Caporaso et al., 2010). Quality filtering of sequences followed Li et al. (2018) using Trimmomatic (Bolger et al., 2014) and Flash (Magoč and Salzberg, 2011). Potential chimeric sequences were detected and removed using *identify chimeric seqs.py* based on the *de novo* method in QIIME. The filtered sequences were then clustered into operational taxonomic units (OTUs) at 97% sequence similarity with UPARSE (Edgar, 2010). OTUs containing a single read (singletons) across all samples were discarded. The taxonomy assignment of OTUs was done using UCLUST against the Protist Ribosomal (PR2) database (Guillou et al., 2012). Bacteria, Archaea, plastidial, and Metazoa affiliated sequences were further removed before downstream analysis.

### Statistical Analyses

To normalize sampling effort, OTU counts were rarefied at the lowest sequence number (9,613). Alpha diversity estimates, including OTU richness, Shannon, and phylogenetic diversity (PD), were calculated using QIIME (Caporaso et al., 2010). Spearman’s rank correlation coefficients were calculated to explore the association between alpha diversity estimates and environmental factors using SPSS v.11.5 (SPSS, Chicago, IL, United States). Beta diversity was calculated with Bray-Curtis and unweighted UniFrac distances and visualized using principal coordinate analysis (PCoA). Similarity percentage (SIMPER) analysis was used to identify OTUs primarily responsible for the differences observed among groupings of samples using Paleontological Statistics (PAST) software (Hammer et al., 2001). Simple and partial Mantel tests (9,999 permutations) were used in R with the Vegan package to explore correlations between environmental parameters and beta diversity (Legendre and Legendre, 1998).

### The Co-occurrence Network Analyses

The 35 samples collected in the present study were separated into two groups, the inner bay group including samples of Z1–Z4 (18 samples) and outer bay group including samples of Z6–Z12 (17 samples), and co-occurrence network analysis was performed, respectively. To reduce the complexity of the data sets, OTUs represented by more than 20 sequences were retained for the construction of networks by SparCC (Friedman and Alm, 2012). Only robust (|*r*| > 0.4) and statistically significant (*p* < 0.01) correlations were incorporated into network analyses. Before that, to reduce the false-positive results, for each network, the values of *p* were adjusted with a multiple testing correction using the Benjamini-Hochberg false discovery rate (FDR) control procedure (Benjamini et al., 2006). Finally, network visualization and module detection were conducted using Gephi software (Bastian et al., 2009).

### Quantification of Ecological Processes

Quantification of ecological process, including selection, dispersal, and drift, was made according to Stegen et al. (2013), which involved two major steps. First, the weighted *ß*-mean nearest taxon distance (*ß*MNTD) was calculated to measure the phylogenetic turnover in order to determine whether communities are under heterogeneous or homogeneous selection (Zhou and Ning, 2017). Null models were then constructed using 999 randomizations as in Stegen et al. (2013). Differences between the observed *ß*MNTD and the mean of the null distribution are denoted as *ß*-Nearest Taxon Index (*ß*NTI), which indicate either the deterministic processes (*ß*NTI >2 or < −2) or stochastic processes (−2 < *ß*NTI < 2) that drives the community assembly. Second, the Bray-Curtis dissimilarities based Raup-Crick metric (RCbray, Chase et al., 2011) was calculated following Stegen et al. (2013) and the randomization was run 999 times. Then, the major ecological processes were partitioned as following: *ß*NTI >2 or <−2 indicate the influence of variable selection and homogeneous selection, respectively. |*ß*NTI| < 2 and RCbray < −0.95 indicate that the community assembly is governed by homogenizing dispersal while RCbray > 0.95 indicate dispersal limitation. In addition, |ßNTI| < 2 and |RCbray| < 0.95 suggest that the community assembly is not dominated by any single process (Stegen et al., 2013).

## Results

### Environmental Factors

The water depth of the sampling sites ranged from 7.7 m at Z6 to 35 m at Z10. Temperature and salinity of the bottom waters ranged from 26.13 to 30.51°C and from 32.62 to 34.19, respectively (Supplementary Table S1). The distribution of environmental parameters was revealed by principal component analysis (PCA; Supplementary Figure S1). Environmental factors of sediments measured showed significant variations among sampling sites (ANOSIM, *R* = 0.456, *p* < 0.001) but were insignificant among sediment depths (ANOSIM, *R* = −0.092, *p* = 0.991).

The concentrations of most metals generally decreased from inner to outer bay, and at each site, the lowest of metal concentrations were usually found at the most in-depth samples (i.e., 5 cm sediment depth). The only exception was Cu, which increased from Z1 to Z8 then decreased toward Z12 (Supplementary Table S1). Slit (4–63 μm) was the major component of sediment grain size fraction at all sites, ranging from 53.8% at Z4 to 81.6% at Z6, followed by sand and clay at most sites (Supplementary Table S1).

### Alpha Diversity and Correlations With Environmental Factors

**Figure 2:**
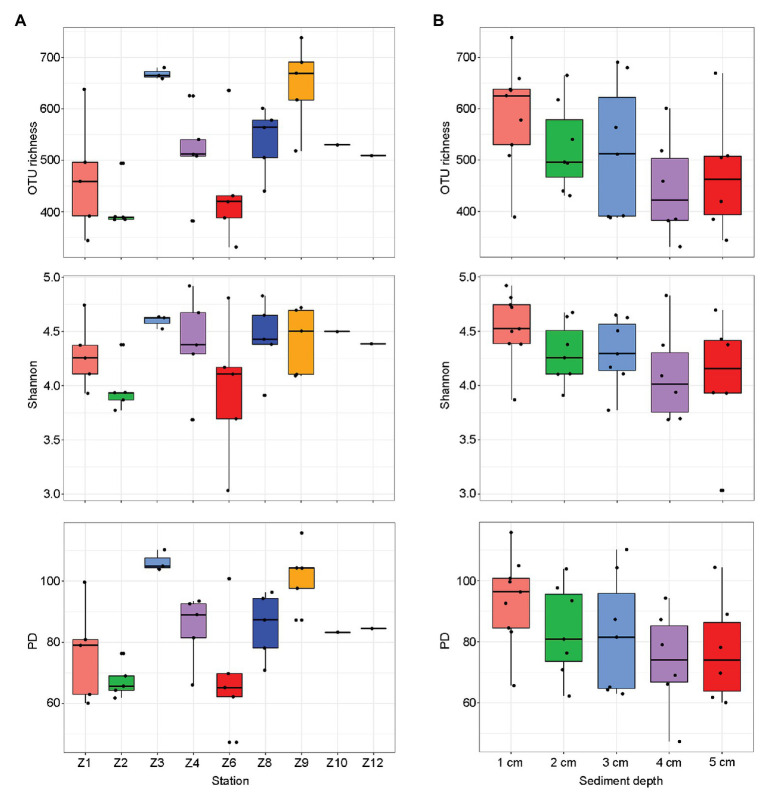
Alpha-diversity estimates [operational taxonomic unit (OTU) richness, Shannon, and phylogenetic diversity (PD)] for each sampling sites **(A)** and sediment depths **(B)**, respectively. The line in each box plot indicates the median, the box delimits the 25th and 75th percentile.

The final dataset contained a total of 2,153,728 reads that were grouped into 409–1,262 OTUs per sample (Supplementary Table S2). After pooling samples from each site, the highest diversity estimates were found at Z9 and the lowest at Z2 (Figure 2A). After pooling samples from the same sediment depth, the diversity estimates decreased with the increasing sediment depth and no significant difference were found (Figure 2B).

Operational taxonomic unit richness had the strongest negative correlation with the proportions of clay (0.1–4 μm; *r* = −0.637, *p* < 0.0001) and positive correlation with the proportions of sand (63–2000 μm; *r* = −0.480, *p* = 0.007), respectively, followed by sediment depth (*r* = −0.427, *p* = 0.011), longitude (*r* = 0.391, *p* = 0.020), latitude (*r* = −0.390, *p* = 0.020), and bottom water temperature (*r* = −0.360, *p* = 0.033; Table 1). Several trace metals had significant correlations with richness, including Cr (*r* = −0.404, *p* = 0.016), Zn (*r* = −0.384, *p* = 0.023), and Pb (*r* = −0.361, *p* = 0.033). Phylogenetic diversity (PD) showed the similar trend with OTU richness while Shannon index only significantly correlated with sediment depth (*r* = −0.397, *p* = 0.018; Table 1).

**Table 1:** Spearman’s correlations between alpha diversity estimates of benthic microbial eukaryotes and environmental variables.

Variable	Richness		Shannon		PD	
	*r*	*p*	*r*	*p*	*r*	*p*
Sediment depth	−0.427	**0.011**	−0.397	**0.018**	−0.383	**0.023**
Longitude	0.391	**0.020**	0.225	0.194	0.360	**0.034**
Latitude	−0.390	**0.020**	−0.224	0.195	−0.360	**0.034**
Bottom water, depth	0.315	0.066	0.088	0.613	0.329	0.054
Bottom water, temperature	−0.360	**0.033**	−0.229	0.185	−0.330	0.053
Bottom water, salinity	0.311	0.069	0.208	0.230	0.297	0.083
Cr	−0.404	**0.016**	−0.319	0.062	−0.375	**0.026**
Cu	0.294	0.086	0.146	0.404	0.276	0.108
Zn	−0.384	**0.023**	−0.262	0.128	−0.409	**0.015**
As	−0.239	0.167	−0.162	0.351	−0.239	0.166
Cd	0.090	0.607	−0.061	0.727	0.054	0.759
Pb	−0.361	**0.033**	−0.256	0.138	−0.352	**0.038**
Hg	0.194	0.265	0.168	0.334	0.111	0.524
Clay (grain size: 0.1–4 μm)	−0.637	**0.000**	−0.350	0.058	−0.655	**0.000**
Silt (grain size: 4–63 μm)	−0.234	0.213	−0.316	0.089	−0.293	0.116
Sand (grain size: 63–2000 μm)	0.480	**0.007**	0.388	**0.034**	0.541	**0.002**

Significant values of *p* (<0.05) are in bold.

**Figure 3:**
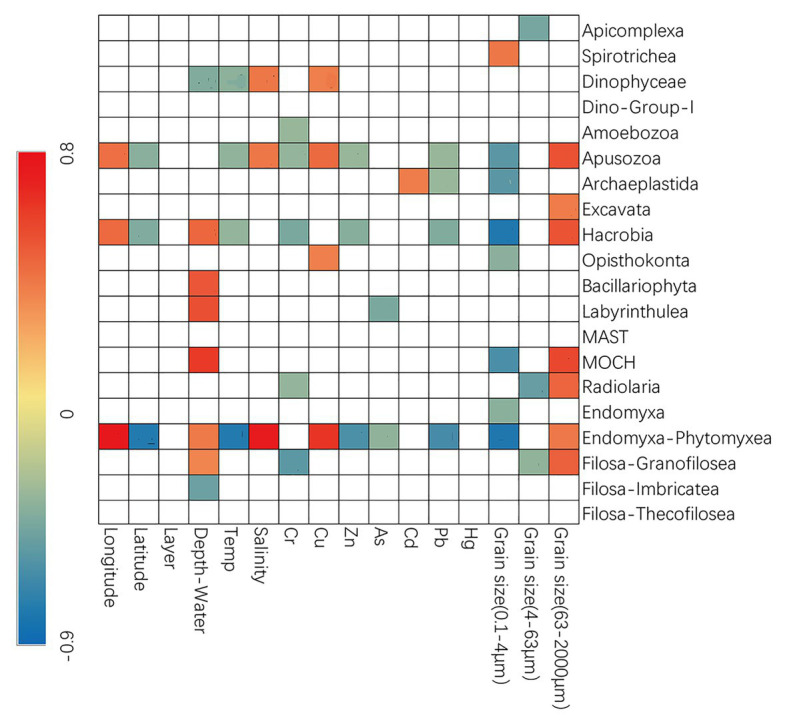
Spearman’s correlations between relative abundance and environmental parameters. The correlation coefficients’ values are indicated according to the color bar and insignificant values (*p* > 0.05) are left blank.

Spearman’s correlation analyses were also conducted to explore the possible influence of environmental variables on the relative sequence abundance of major taxonomic groups (Figure 3). The fraction of sand was usually positively correlated with several groups of microbial eukaryotes (e.g., Apusozoa, Excavata, Hacrobia, MOCH, Radiolaria, Endomyxa-Phytomyxea, etc.) while that of clay and silt (0.1–4 and 4–63 μm) usually had the negative effects. Dinophyceae, Apusozoa, Opisthokonta, and Endomyxa-Phytomyxea were more abundant at sites with higher concentrations of Cu while Zn, As, and Pb had negative influences (Figure 3).

### Beta Diversity and Its Driving Factors

**Figure 4:**
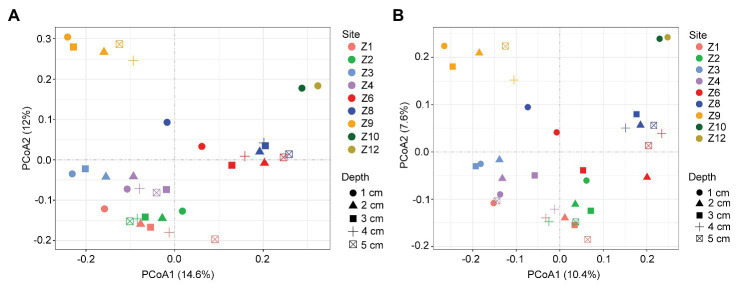
Plots of principal coordinates analysis (PCoA) of microbial eukaryotes based on Bray Curtis dissimilarities **(A)** and Unweighted UniFrac distance matrices **(B)**.

In the two-dimensional principle component analysis (PCoA) of microbial eukaryotes based on Bray-Curtis distance, samples were clustered into four groups. Group_1 included samples from Z1 to Z4. Group_2 included samples of Z6 and Z8. Group_3 included samples of Z9, and Group_4 included samples of Z10 and Z12 (Figure 4A). This clustering pattern was also supported by the PCoA plot of community taxonomic relatedness quantified by the unweighted UniFrac metric (Figure 4B). The grouping pattern was statistically supported (ANOSIM, *R* = 0.768, *p* < 0.001 in Bray Curtis and *R* = 0.699, *p* < 0.001 in unweighted Unifrac metrics, respectively).

Similarity percentage analysis identified 20 OTUs, each of which contributed >1% of the dissimilarities in the microbial eukaryotic communities among the four groups. These OTUs were affiliated with Ciliophora (seven OTUs, mostly members of Spirotrichea), Bacillariophyta (six OTUs, mostly members of Mediophyceae), Dinophyceae (three OTUs), the parasitic Syndiniales (three OTUs), and Cercozoa (one OTU; Supplementary Table S3). The cumulative contributions of all OTUs of a given taxon showed that OTUs affiliated with Bacillariophyta contribute the most (ca. 25.5%) to the dissimilarities among the four groups, followed by Dinophyceae (ca. 21.9%) and Spirotrichea (ca. 16.9%). The rest groups each contributed <5% to the differences among the four groups (Supplementary Table S4).

In the simple Mantel test, geographic distance, water depth, and environmental factors including temperature and salinity of bottom water, the concentration of Cu, and grain size compositions of the sediments were significantly correlated with the microbial eukaryotic communities (Table 2). The partial Mantel test showed that geographic distance made a slightly greater contribution to community variation, with higher *R* values of geographic distance (0.428 after control for water depth and 0.655 after control for environment, respectively) than those of water depth (0.336 after control for geographic distance and 0.611 after control for environment) and environment (−0.157 after control for geographic distance and 0.121 after control for water depth; Table 2). These results were also supported in the plots of geographic distance, water depth, and environmental heterogeneity vs. community similarity, respectively, all of which had negative slopes, with geographic distance showing the highest correlation (Supplementary Figure S2).

**Table 2:** Simple and partial Mantel tests for the correlations between spatial/environmental factors and benthic microbial eukaryotes communities.

Mantel test			Partial Mantel test control for		
	*R*	*p*		*R*	*p*
Geographic distance	0.673	<**0.001**	Water depth	0.428	<**0.001**
			Environment	0.655	<**0.001**
Environment	0.256	**0.008**	Water depth	0.121	0.244
			Geographic distance	−0.157	0.155
Water depth	0.637	<**0.001**	Environment	0.611	<**0.001**
			Geographic distance	0.336	<**0.001**
Sediment depth	0.038	0.244			
Bottom water, temperature	0.466	<**0.001**			
Bottom water, salinity	0.203	0.**006**			
Cr	0.092	0.148			
Cu	0.310	<**0.001**			
Zn	0.021	0.376			
As	0.092	0.155			
Cd	−0.030	0.606			
Pb	0.076	0.194			
Hg	−0.046	0.663			
Clay (grain size 0.1–4 μm)	0.344	<**0.001**			
Silt (grain size 4–63 μm)	0.101	0.134			
Sand (grain size 63–2000 μm)	0.182	**0.031**			

Numbers in bold indicate statistically significant results.

### Community Composition

Overall, the benthic microbial eukaryotic communities of the Dongshan Bay were dominated by Alveolata (ca. 48% of total sequences), followed by Stramenopiles (ca. 37%) and Rhizaria (ca. 10%). The other groups, including Amoebozoa, Apusozoa, Archaeplastida, Excavata, Opisthokonta, and Hacrobia contributed only minorly (Supplementary Figure S3A). Within Alveolata, the major components were Ciliophora (ca. 21% of total sequences), followed by Dinophyceae (ca. 18%), the parasitic groups Dino-Group-I (ca. 6%) and Apicomplexa (ca. 3%), and the rest taxa. Within Stramenopiles, Bacillariophyta accounted for ca. 34% of total sequences.

**Figure 5:**
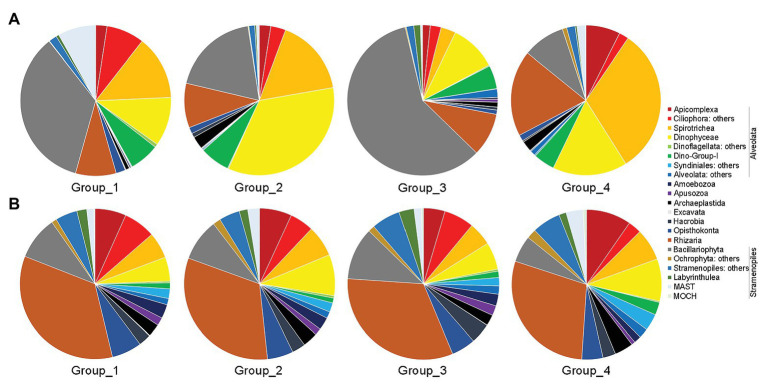
Overview of relative abundance of sequences **(A)** and OTUs richness **(B)** of major benthic microbial eukaryotes assemblages among the four groups. Group_1, samples of Z1–Z4; Group_2, samples of Z6 and Z8; Group_3, samples of Z9; and Group_4, samples of Z10 and Z12.

Group_1 was dominated by Bacillariophyta (ca. 35% of total sequences), followed by Ciliophora (ca. 22%), Dinophyceae (ca. 11%), Rhizaria (ca. 9%), and the rest groups (Figure 5A). Dinophyceae surpassed Bacillariophyta to be the most dominant group in Group_2, accounting for ca. 34% of all sequences, followed by Ciliophora (ca. 20%), Bacillariophyta (ca. 19%), Rhizaria (ca. 10%), Dino-Group-1 (ca. 6%), and the rest groups. Group_3 was characterized by high contributions of Bacillariophyta (ca. 59%), followed by Dinophyceae (ca. 10%), Rhizaria (9%), Dino-Group-1 (ca. 5%), and other lineages. Within Group_4, Ciliophora was the top contributor (ca. 34%), followed by Rhizaria (ca. 19%), Dinophyceae (ca. 16%), Bacillariophyta (ca. 9%), and other groups (Figure 5A). Cercozoa mainly comprised members affiliated with Filosa-Imbricatea in Group_1 and Group_2, Filosa-Thecofilosea in Group_3, and Endomyxa-Phytomyxea in Group_4 in terms of relative sequence abundances, respectively (Supplementary Figure S4A).

In terms of OTU richness, Alveolata and Rhizaria were the highest contributors, and each contributing ca. 33% of total OTUs counts, followed by Stramenopiles (ca. 19%) and Opisthokonta (ca. 6%; Figure 5B). Each of the rest groups contributed on average less than 5% of total OTUs. Within Alveolata, Ciliophora was the major contributor (ca. 12%), followed by Dinophyceae (ca. 7%) and the rest groups. The unclassified Cercozoa, Filosa-Thecofilosea, and Filosa-Imbricatea were the major components of Cercozoa (Supplementary Figure S4B). Within Stramenopiles, OTUs affiliated with Bacillariophyta contributed the most (ca. 9%). The OTUs compositions differed slightly among the four groups (Figure 5B).

### Co-occurrence Networks

**Figure 6:**
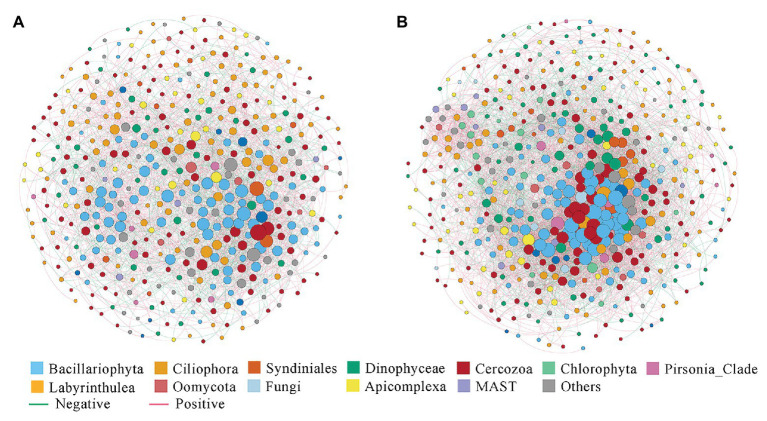
Co-occurrence networks of microbial eukaryotes OTUs in the inshore **(A)** and offshore **(B)** sediments of the Dongshan Bay.

The correlation-based network consisted of 449 nodes (OTUs) and 2,759 edges (correlations) for the nearshore group, and 500 nodes and 6,483 edges for the offshore group (Figure 6). Several parameters representing the network topological structure were calculated. The degree, network diameter, density, and average clustering coefficient appeared to be higher in the offshore network compared to the nearshore network, whereas the modularity and average path length showed an inverse trend (Supplementary Table S5). These results indicated that microbial eukaryotic OTUs were more connected in offshore than in nearshore sediments. In the meantime, higher positive edges among OTUs were found in nearshore than in offshore network (62.02 vs. 58.04%). Comparisons among some microbial eukaryotic groups showed noticeable changes of inter microbial eukaryotes relationships between the nearshore and offshore environments. For example, the position correlations between Syndiniales and Dinophyceae decreased from 72.7% in the nearshore group to 34.85% in the offshore group. The positive correlations between Cercozoa and Bacillariophyta decreased from 55.89% in the nearshore group to 23.87% in the offshore group while those between Cercozoa and Dinophyceae increased from 48.86 to 76.54% (Supplementary Table S6).

### Community Assembly Processes

**Figure 7:**
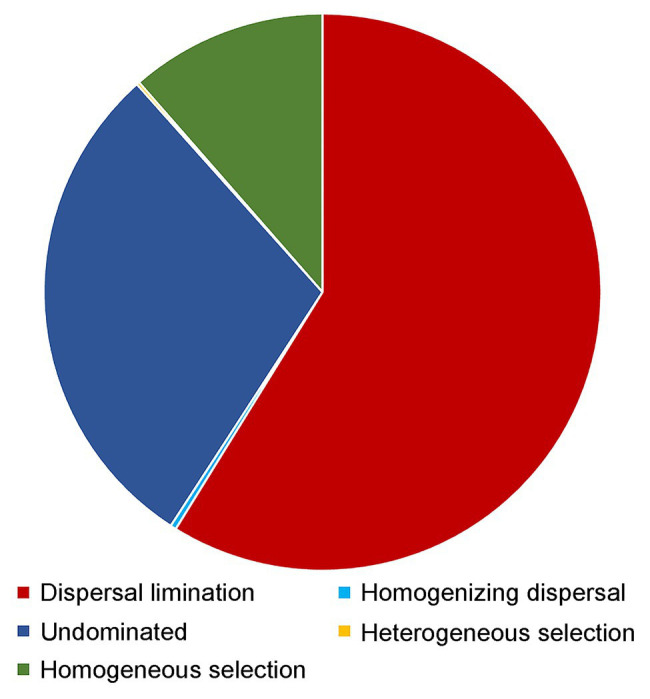
Partition of the community assembly process of benthic microbial eukaryotes.

Dispersal limitation was found to be the primary driver for the community assembly processes of benthic microbial eukaryotes and explained 58.8% of community turnover, followed by drift (ca. 29.2%) and homogeneous selection (ca. 11.4%). The rest processes, including homogenizing selection and heterogeneous selection, totally accounted for ca. 0.5% of community turnover (Figure 7).

## Discussion

### The Predominance of Alveolata, Rhizaria, and Stramenopiles Affiliated Sequences/OTUs in the Benthic Microbial Eukaryotic Communities

Since the pioneering work of López-García et al. (2001) and Moon-van der Staay et al. (2001), there has been a surge of studies on microbial eukaryotes diversity using sequencing based approaches from diverse marine environments, focusing more on the water columns than sediments (Edgcomb et al., 2002; Countway et al., 2007; Not et al., 2009; Scheckenbach et al., 2010; Pernice et al., 2015; Flegontova et al., 2016; Xu et al., 2018). Although large variabilities were found in the community compositions of marine pelagic microbial eukaryotes, they are usually dominated by Alveolata and Stramenopiles in term of relative sequence abundance both in the coastal and open oceanic waters (e.g., Countway et al., 2007; de Vargas et al., 2015; Massana et al., 2015; Pernice et al., 2015). The contribution of Rhizaria (represented mainly by Radiolaria) to the total microbial eukaryotic communities generally increased with the increasing water depth in the DNA dataset although a smaller portion was found in the RNA extractions (Not et al., 2009; Xu et al., 2017a). In term of OTU richness, the sunlit layer of the ocean was found to be represented by diplonemids (Excavata), which sometimes surpassed other groups to be the most dominant group (Flegontova et al., 2016). Molecular studies on the marine benthic microbial eukaryotes have largely lagged behind of those of marine plankton and direct comparisons of benthic microbial eukaryotic communities with overlying water masses are very rare. The very sparse and limited studies showed little overlap between benthic and planktonic communities of microbial eukaryotes and microbial eukaryotes in the sediments showed high variations even at microscale (Doherty et al., 2010; Forster et al., 2016). Habitat heterogeneity in marine sediments caused by grain size composition, dissolved oxygen, organic matter composition, and food availability, which varied significantly between sediments and overlaying seawater was proposed to be the major reasons for the difference of microbial eukaryotic communities found between these two major marine habitats (Pedersen et al., 2015; Forster et al., 2016). One of the striking difference between the microbial eukaryotes dwelling in the sediment and water column is the dominance of Cercozoa affiliated OTUs in the marine sediments (Pawlowski et al., 2011; Wu and Huang, 2019). In our study, ca. 29% of all OTUs recovered was affiliated with Cercozoa (Supplementary Figures S3, S4). In soil/sediment, Cercozoa has long been recognized as major components of predatory protozoa (Bates et al., 2013; Geisen et al., 2015; Harder et al., 2016; Fiore-Donno et al., 2019). On the deep-sea floor, Cercozoa was found to comprise on average 16% of all eukaryote affiliated OTUs (Pawlowski et al., 2011). In a recent study, Wu and Huang (2019) reported that Cercozoa dominated protistan community in surface seafloor sediments of the South China Sea, accounting for ca. 40 and 25% of total sequences and OTUs richness, respectively. The prevailing and sometime dominance of Cercozoa affiliated taxa found in the present and previous studies indicated that they might play potentially pivotal roles in both the coastal and deep-sea sediments, which is worth more detailed exploration in future studies.

Our results showed that Alveolata predominated the microbial eukaryotic communities in the coastal sediments of the Dongshan Bay in terms of both relative sequences abundance (ca. 57%) and OTUs richness (ca. 36%; Supplementary Figure S3). This is consistent with previous studies on other coastal regions (Chen et al., 2017; Zhang et al., 2018; Kong et al., 2019). Previous morphology-based surveys have shown that many dinoflagellates can form cysts as a part of their life cycle, which could remain viable for several years in the sediments (Lewis et al., 1999). Previous studies also showed that Ciliophora dominated microbial eukaryotic communities in various marine sediment environments (Doherty et al., 2010; Gong et al., 2015; Massana et al., 2015; Pasulka et al., 2016; Zhao and Xu, 2016, 2017; Pan et al., 2020), which is constant with the present study showing that Ciliophora, represented mainly by Class Spirotrichea, predominated across all samples, accounting for ca. 21% of total sequences and 10% of total OTUs counts, respectively. Class Spirotrichea comprises morphologically diverse groups of ciliates including naked oligotrichs and tintinnids that sometimes dominated microzooplankton community in both coastal areas and open ocean (Lynn, 2008; Sun et al., 2017, 2020; Huang et al., 2020; Xu et al., 2020; Yang et al., 2020). Based on the present and previous studies, this group of ciliates may play an important yet unclear ecological function in marine sediments.

Our study showed that Bacillariophyta accounted for ca. 34% of total sequences and ca. 20% of OTU richness. Kong et al. (2019) found that Bacillariophyta dominated the intertidal microbial eukaryotic communities (ca. 92.0 ± 2.2% of sequences and ca. 65.8 ± 2.7% of OTU richness). The dominance of Bacillariophyta affiliated sequences have also been reported in other studies on marine intertidal and coastal sediment environments using both sequencing (Gong et al., 2015; Massana et al., 2015; Pan et al., 2020) and microscopy/pigment (Baillie, 1987; Sullivan and Moncreiff, 1988; Cartaxana et al., 2006; Sahan et al., 2007; Schüller and Savage, 2011) based techniques.

### Controlling Factors of the Alpha Diversity of Benthic Microbial Eukaryotes

Our study showed that the propotion of 0.1–4 μm grain size (clay) of the sediment had the strongest negative correlation with the alpha diversity estimates while that of the 63–2,000 μm grain size fraction (sand) had the strongest positive correlation, respectively. It has been previously shown that the benthic prokaryotic community was driven partially by the grain size composition of the sediment (Dang et al., 2010). Benthic microbial eukaryotes are composed of heterotrophic groups, including ciliates and heterotrophic nanoflagellates, that prey on prokaryotes (Fenchel, 1978; Pasulka et al., 2017). Thus, the changes of prokaryotic communities due to grain size composition might further influence the grazers. In the meantime, it may be probably due to the fact that some microbial eukaryotes groups (e.g., ciliates and cercozoans) need space between sediment grains for locomotion (Zhao and Xu, 2017; Zhu et al., 2017; Kong et al., 2019).

Our study also showed that sediment depth had a strong correlation (*R* = −0.427, *p* = 0.011) with the alpha diversity estimates, and the sediment depth decay pattern of alpha diversity estimates was indeed found in Shannon and OTU richness (Supplementary Figure S5B). Environmental DNA based sequencing was used in the present study. Thus, the DNA sequences retrieved revealed the “total” microbial eukaryotic community, which may include live, dead, dormant cells, and even extracellular free DNA. Sediment depth also reflects the change of deposition rate and sediment nature (Pietramellara et al., 2009). It is known that the preservation of DNA is affected by the mineral composition of the sediment matrix and the nature and quantity of organic matter in the marine sediments (Pietramellara et al., 2009). So, the preservation and decay of DNA in each layer might also cause the changes in the alpha diversity estimates.

The concentrations of metal including Cr, Zn, and Pb were found to be negatively correlated with the alpha diversity estimates. Gong et al. (2015) reported that in the Yellow Sea, the alpha diversity of the benthic microbial eukaryotes was negatively correlated with Zn. Kong et al. (2019) reported that the alpha diversity of the intertidal microbial eukaryotes significantly correlated with Cd, Cu, and Zn. It has been reported that high concentrations of heavy metals can alter the soil bacterial communities (He et al., 2017; Kasemodel et al., 2019). The proportion of anthropogenic Pb has been reported to increase from 9 to 15% during 2000–2014 on the coast surrounding Dongshan Island (Xu et al., 2017b). Therefore, we can speculate that the benthic microbial eukaryotic community in Dongshan Bay has undergone great changes in recent years. Unfortunately, no time-series sampling was performed; thus, the changes of benthic microbial eukaryotes in Dongshan Bay responding to the increasing concentrations of metals cannot be achieved now.

Previous studies have showed a water depth gradient of the alpha diversity for benthic microbial eukaryotes (Bik et al., 2012; Gong et al., 2015), which was not confirmed in our study. The depths of the sampling sites in the present study ranged from 7.7 to 35 m, and seawater may be relatively well mixed due to tide and other physical forces and may not exert enough screening pressure on the alpha diversity of benthic microbial eukaryotes.

### Controlling Factors of the Beta Diversity of Benthic Microbial Eukaryotes

Previous studies found that water depth can strongly influence the benthic protistan communities both in the deep sea and coastal sediments (Bik et al., 2012; Gong et al., 2015; Wu and Huang, 2019), which is also confirmed by the present study (Table 2). Water depth as a key factor structuring the community of benthic microbial eukaryotes could be due to the fact that it may serve as a proxy for many physical and chemical variables including water temperature, salinity, pressure, nutrients, light, dissolved organic carbon, etc. Interestingly, sediment depth was found to have no significant correlation with the beta diversity of the microbial eukaryotic community in the present study (Table 2). A previous study reported that sediment in the Dongshan Bay underwent mixing or bioturbation in the upper 5 cm sediment which may cause the facts seen in the present study (Xu et al., 2017b).

Our study found that after controlling for environmental parameters using partial Mantel test, geographic distance still showed a significant correlation with beta diversity, which indicated a high spatial variation of microbial eukaryotic communities in the sediments. A distance-decay relationship of the community composition of microbial eukaryotes was indeed found in the present study (Supplementary Figure S2B). In fact, the distance decay pattern of microbial communities including both prokaryotes and eukaryotes was reported in previous studies in both the pelagic and benthic environments (Scheckenbach et al., 2010; Jacob et al., 2013; Gong et al., 2015; Sun et al., 2020; Wu et al., 2020). The distance decay pattern of microbial communities may indicate the dispersal limitation and selection process of microbes (Hanson et al., 2012), which is especially true for benthic microbial groups because the movements of microbial eukaryotes are more restricted in the sediments than in water column due to the nature of the sediment proposition. The PCoA analysis on environmental parameters measured in the present study showed that samples were clustered basically according to the location of the sampling sites, i.e., the sampled benthic habitats are strongly different (Supplementary Figure S1; ANOSIM, *R* = 0.456, *p* < 0.001). Null model analysis showed that dispersal limitation accounted for ca. 59% of microbial eukaryotic communities followed by drift (ca. 29%) and homogeneous selection (ca. 11%), which may help explain the results found in the study (Figure 7). Dispersal limitation dominated the community assembly process of microbial eukaryotes was also reported in deep-sea sediments and oceanic sea waters (Logares et al., 2018; Wu and Huang, 2019; Sun et al., 2020; Xu et al., 2020), which indicates the prevailing of dispersal limitation as a major driving force of microbial eukaryotic communities in various marine environments.

Co-occurrence network analysis between the nearshore and offshore environments showed changes of inter microbial eukaryotes relationships among some groups. For example, the positive correlations between Syndiniales and Dinophyceae decreased from 72.73% in nearshore to 34.85% in offshore sediments. Syndiniales have long been known as a ubiquitous group of protist parasites including dinoflagellates, ciliates, and radiolarians (Guillou et al., 2008). Previous studies have shown that many factors including host density, water temperature, nutrient, water depth, etc., can influence the dynamics of Syndiniales populations, among which host density was identified as the major driver (Coats et al., 1996; Park et al., 2004). Unfortunately, we did not enumerate the abundances of Dinophyceae in the sediments of Dongshan Bay. It has been shown that some cysts of Dinophyceae were primarily associated with sediments dominated by mud (Nehring, 1994). In our study, we did find that the content of mud in the nearshore sites were higher than that in the offshore sites (Supplementary Table S1). The decreasing of mud proportions from nearshore to offshore sediments may influence the density of benthic Dinophyceae. The decreasing abundances of Dinophyceae may lead to lower encounters and infection of hosts by Syndiniales which may be reflected by the dropping of positive correlations in the offshore environment than in the nearshore environment. The network analysis also found that the positive correlations between Cercozoa and Bacillariophyta decreased from 55.89% in the nearshore group to 23.87% in the offshore group while those between Cercozoa and Dinophyceae increased from 48.86 to 76.54% (Supplementary Table S6). Cercozoa has been previously identified as a group of protozoa that are capable of being parasites or grazers (Geisen et al., 2015; Harder et al., 2016). They could function as diatoms (or dinoflagellates) grazers and parasites and cause the population dynamic of diatoms/dinoflagellates (Tillmann et al., 1999; Schnepf and Kuhn, 2000; Genitsaris et al., 2015). Again, due to the unavailability of the abundances of dinoflagellates and diatoms in our samples, we can only assume that the changes of the correlations between Cercozoa and Dinophyceae/Bacillariophyta in the nearshore and offshore sediments could be caused by the changes of the community composition/abundances of the Dinophyceae/Bacillariophyta lineages. More data including detailed information on the abundances/community composition of Dinophyceae/Bacillariophyta groups along the transect using microscopy based techniques as well as RNA based sequencing approaches will shed more light on the interactions among these groups in the sediments.

### Conclusion

Out study showed that the sediment microbial eukaryotes were dominated by Alveolata (mainly Ciliophora and Dinophyceae) in terms of relative sequences abundance followed by Stramenopiles (mainly Bacillariophyta) and Rhizaria (mainly Cercozoa). While in terms of OTU richness, Alveolata and Rhizaria contributed equally, being the two most abundant groups. Distinct driving factors of the alpha and beta diversities of benthic microbial eukaryotes were found. Grain size composition, sediment depth, and heavy metal including Cr, Zn, and Pb were identified to drive the alpha diversity of microbial eukaryotes while water depth showed no significant effect. Geographic distance and water depth surpassed other environmental factors to be the primary factors shaping the benthic microbial eukaryotic communities. Disclosing the spatial distribution patterns and shaping factors of benthic microbial eukaryotes in a bay highly influenced by human activities will be helpful to understand the response of sediment microbial communities to environmental changes.

## Data Availability Statement

The datasets presented in this study can be found in online repositories. The names of the repository/repositories and accession number(s) can be found at: https://www.ncbi.nlm.nih.gov/genbank/, PRJNA645757.

## Author Contributions

DX and PS conceived and designed the study. RG collected the samples and conducted the experiments. RG, YW, PS, and DX analyzed the data. FY performed the grain size analysis. All authors contributed to the article and approved the submitted version.

### Conflict of Interest

The authors declare that the research was conducted in the absence of any commercial or financial relationships that could be construed as a potential conflict of interest.
