# Supplementary material for: Genetic Diversity, Community Assembly, and Shaping Factors of Benthic Microbial Eukaryotes in Dongshan Bay, Southeast China
Source: Front Microbiol. 2020 Dec 23;11:592489. doi: 10.3389/fmicb.2020.592489 (PMC7785585; doi:10.3389/fmicb.2020.592489)

**FIGURE S1** Principal component analysis (PCA) showing environmental variations of all samples in the Dongshan Bay.


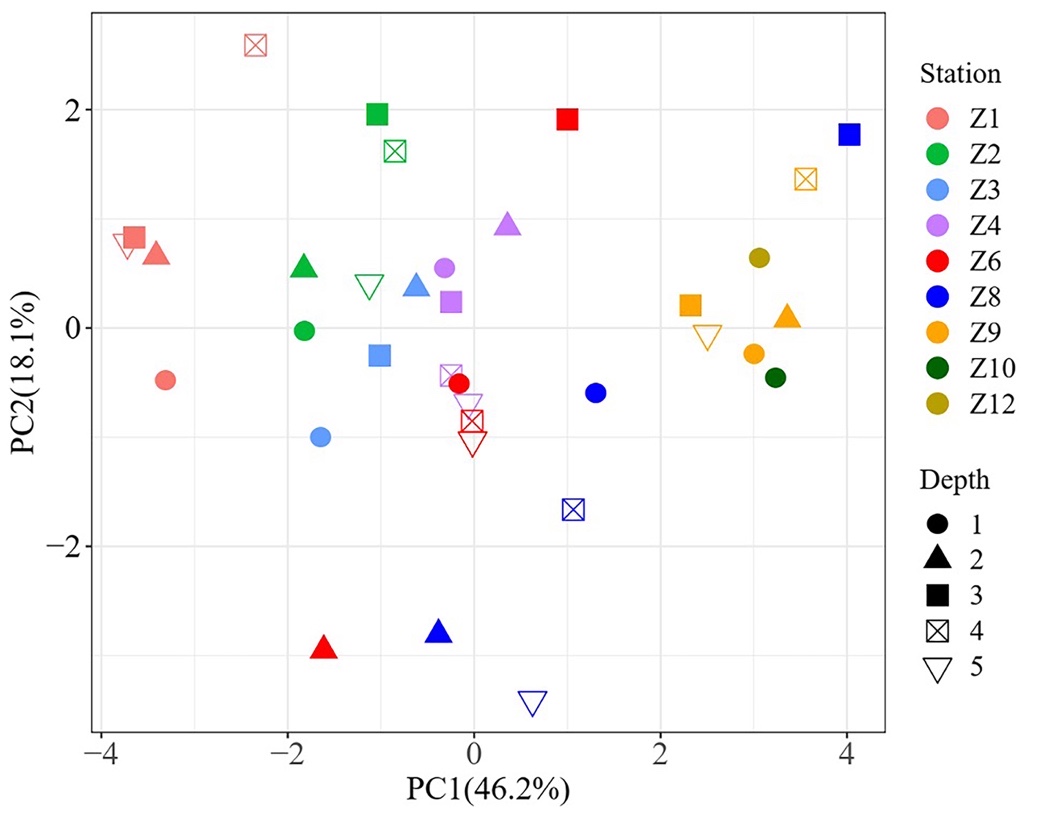


**FIGURE S2.** Correlation of community similarity with geographic distance (A), water depth (B), and environmental distance (C).


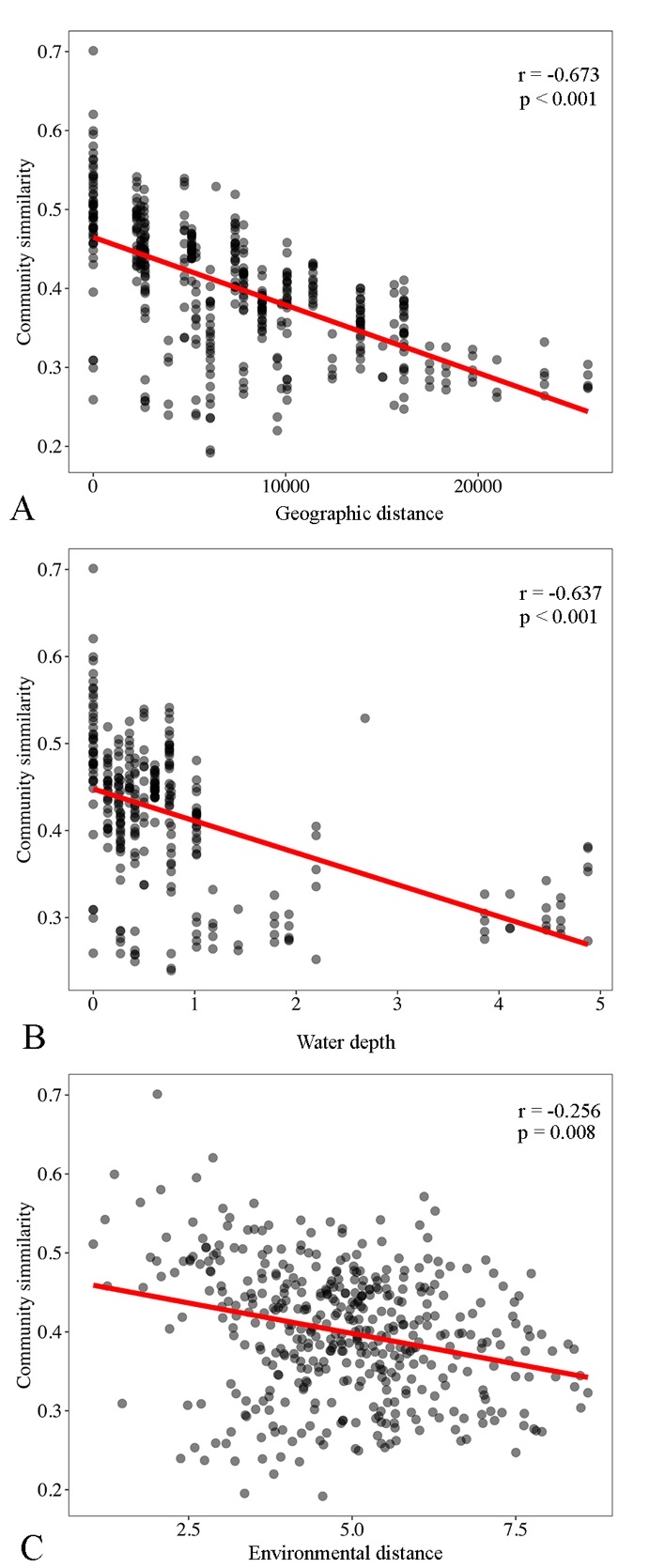


**FIGURE S3** Overview of the proportional sequence number (A) and OTUs richness (B) of major assemblages of benthic microbial eukaryotes in the Dongshan Bay.


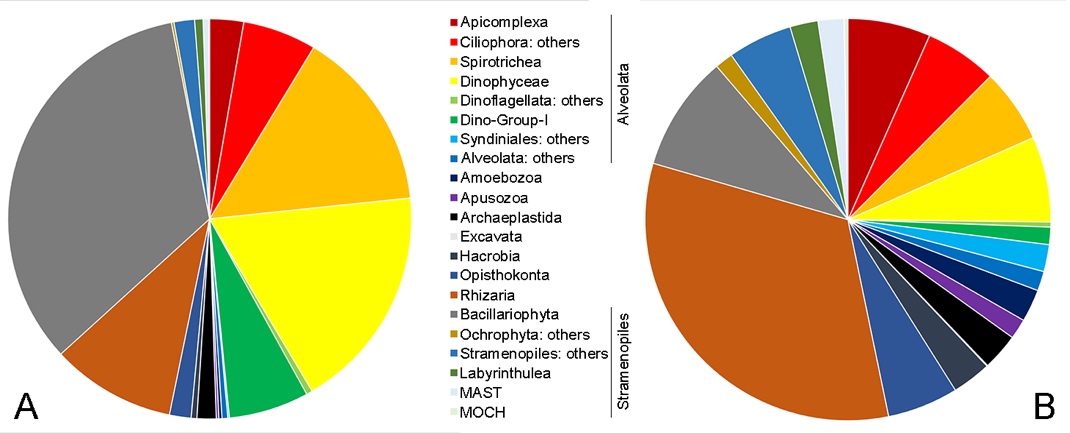


**FIGURE S4** Overview of the proportional sequence number (A) and OTUs richness (B) of major lineages of Rhizaria in the four groups identified in **Figure 4**.


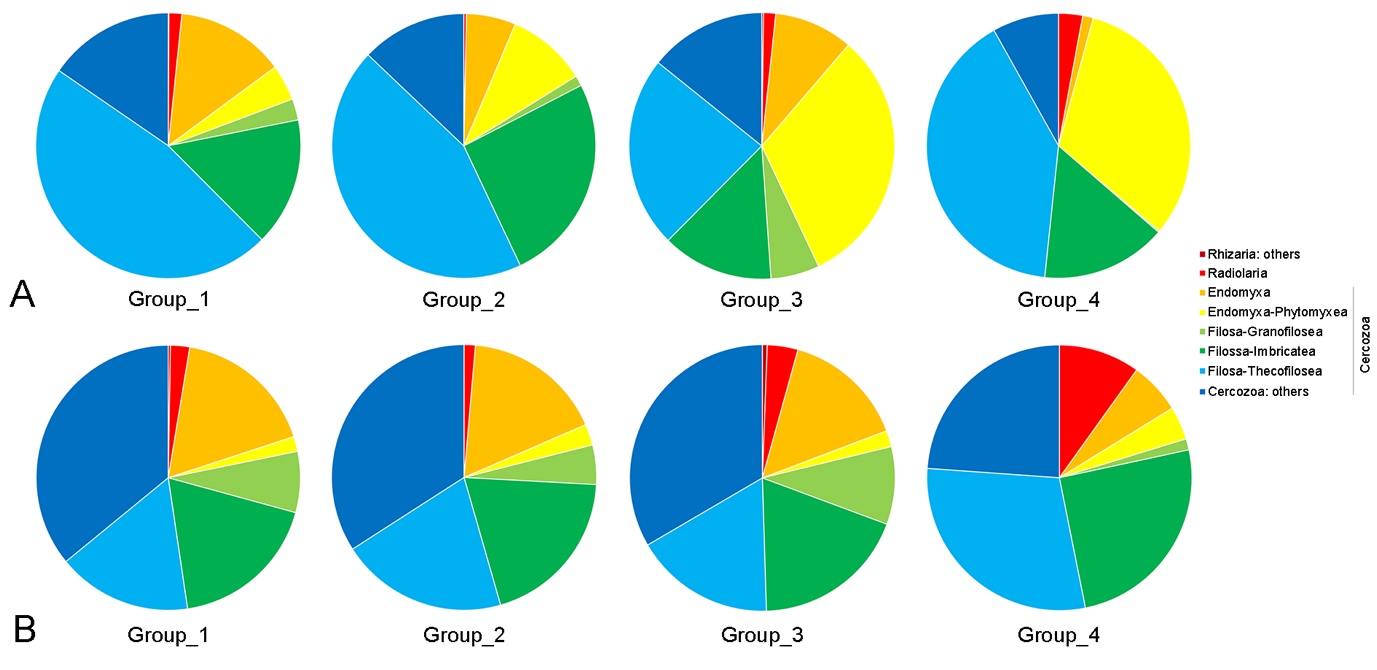


**FIGURE S5** Correlation of alpha diversity estimates including OTUs richness (A), shannon (B), and PD (C) with sediment depth.


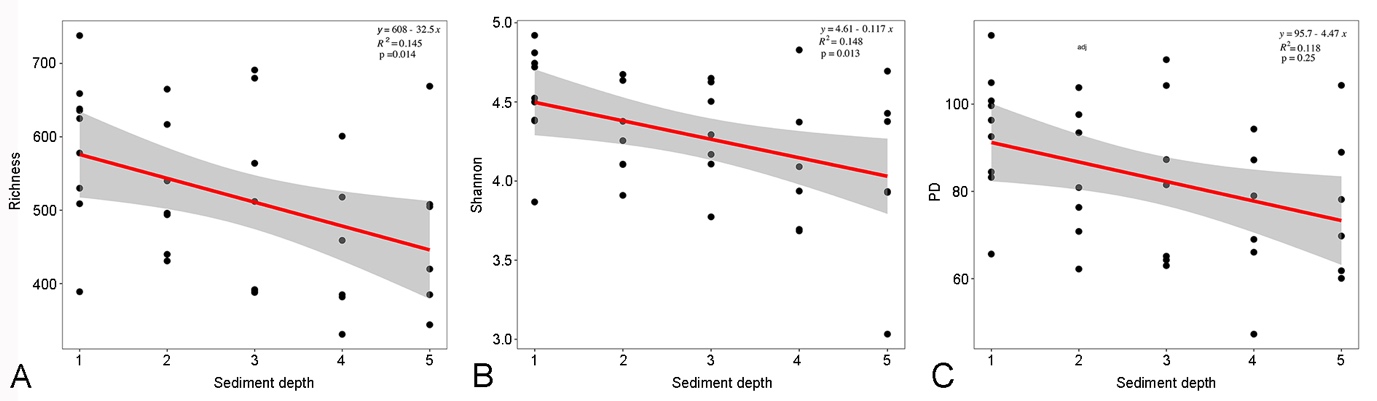

Supplement: Supplementary file 1 [file Data_Sheet_1.zip › Supplemental figures.docx]
