# Supplementary material for: Genetic Diversity, Community Assembly, and Shaping Factors of Benthic Microbial Eukaryotes in Dongshan Bay, Southeast China
Source: Front Microbiol. 2020 Dec 23;11:592489. doi: 10.3389/fmicb.2020.592489 (PMC7785585; doi:10.3389/fmicb.2020.592489)
Supplement: Supplementary file 1 [file Data_Sheet_1.zip › Table_S2.docx]

**TABLE S2** Alpha diversity estimates of Dongshan Bay samples. OTU_0.03_, Operational taxonomic unit at 97% SSU rRNA V4 gene sequence identity. *OTU before normalization. **Standardized numbers based on subsampling of 9,613 sequences without replacement.

| Sample ID | OTU_0.03_* | OTU_0.03_** | Shannon** | PD** |
| --- | --- | --- | --- | --- |
| Z1-1 | 902 | 642 | 4.75 | 99.63 |
| Z1-2 | 933 | 499 | 4.28 | 80.89 |
| Z1-3 | 641 | 397 | 4.12 | 62.99 |
| Z1-4 | 767 | 464 | 4.38 | 79.02 |
| Z1-5 | 412 | 345 | 3.93 | 60.11 |
| Z2-1 | 662 | 392 | 3.87 | 65.66 |
| Z2-2 | 907 | 498 | 4.38 | 76.35 |
| Z2-3 | 642 | 392 | 3.77 | 64.31 |
| Z2-4 | 635 | 387 | 3.94 | 69.02 |
| Z2-5 | 568 | 389 | 3.94 | 61.84 |
| Z3-1 | 1247 | 663 | 4.54 | 104.91 |
| Z3-2 | 1262 | 669 | 4.65 | 103.80 |
| Z3-3 | 1175 | 682 | 4.63 | 110.19 |
| Z4-1 | 869 | 625 | 4.92 | 92.56 |
| Z4-2 | 540 | 540 | 4.67 | 93.45 |
| Z4-3 | 897 | 514 | 4.3 | 81.51 |
| Z4-4 | 495 | 383 | 3.69 | 66.08 |
| Z4-5 | 775 | 508 | 4.38 | 88.98 |
| Z6-1 | 1098 | 637 | 4.81 | 100.76 |
| Z6-2 | 674 | 431 | 4.11 | 62.22 |
| Z6-3 | 517 | 389 | 4.17 | 65.17 |
| Z6-4 | 409 | 332 | 3.7 | 47.32 |
| Z6-5 | 557 | 421 | 3.04 | 69.79 |
| Z8-1 | 958 | 578 | 4.38 | 96.33 |
| Z8-2 | 752 | 442 | 3.91 | 70.85 |
| Z8-3 | 1025 | 565 | 4.65 | 87.31 |
| Z8-4 | 976 | 601 | 4.83 | 94.29 |
| Z8-5 | 876 | 509 | 4.43 | 78.18 |
| Z9-1 | 1195 | 738 | 4.72 | 115.75 |
| Z9-2 | 1084 | 618 | 4.11 | 97.61 |
| Z9-3 | 1163 | 692 | 4.5 | 104.26 |
| Z9-4 | 745 | 518 | 4.09 | 87.22 |
| Z9-5 | 1018 | 671 | 4.7 | 104.31 |
| Z10 | 812 | 530 | 4.5 | 83.24 |
| Z12 | 835 | 511 | 4.39 | 84.47 |
