# Supplementary material for: Genetic Diversity, Community Assembly, and Shaping Factors of Benthic Microbial Eukaryotes in Dongshan Bay, Southeast China
Source: Front Microbiol. 2020 Dec 23;11:592489. doi: 10.3389/fmicb.2020.592489 (PMC7785585; doi:10.3389/fmicb.2020.592489)
Supplement: Supplementary file 1 [file Data_Sheet_1.zip › Table_S3.docx]

**TABLE S3** Taxonomic identities of the 20 OTUs that contributed most to community dissimilarities among the four groups with their relative contributions to community dissimilarities.

| OTU ID | Taxonomy identity | Contribution to dissimilarity (%) | Cumulative contribution (%) |
| --- | --- | --- | --- |
| OTU_4 | Stramenopiles: Ochrophyta: Bacillariophyta: Bacillariophyta_X: Polar-centric-Mediophyceae | 4.875 | 4.875 |
| OTU_9 | Alveolata: Dinoflagellata: Dinophyceae: Peridiniales: Diplopsalidaceae | 4.21 | 9.086 |
| OTU_21 | Alveolata: Dinoflagellata: Dinophyceae | 2.474 | 11.56 |
| OTU_11 | Stramenopiles: Ochrophyta: Bacillariophyta: Bacillariophyta_X: Polar-centric-Mediophyceae | 1.773 | 13.33 |
| OTU_5 | Stramenopiles: Ochrophyta: Bacillariophyta: Bacillariophyta_X: Polar-centric-Mediophyceae | 1.722 | 15.05 |
| OTU_15 | Stramenopiles: Ochrophyta: Bacillariophyta: Bacillariophyta_X: Polar-centric-Mediophyceae: Skeletonema: *Skeletonema marinoi* | 1.61 | 16.66 |
| OTU_16 | Stramenopiles: Ochrophyta: Bacillariophyta: Bacillariophyta_X: Polar-centric-Mediophyceae: *Chaetoceros* | 1.57 | 18.23 |
| OTU_87 | Alveolata: Ciliophora: Spirotrichea: Strombidiida | 1.536 | 19.77 |
| OTU_57 | Stramenopiles: Ochrophyta: Bacillariophyta: Bacillariophyta_X: Raphid-pennate | 1.429 | 21.2 |
| OTU_6 | Alveolata: Dinoflagellata: Syndiniales: Dino-Group-I: Dino-Group-I-Clade-4: Dino-Group-I-Clade-4_X: Dino-Group-I-Clade-4_X_sp. | 1.387 | 22.59 |
| OTU_12 | Alveolata: Ciliophora: Spirotrichea | 1.365 | 23.95 |
| OTU_28 | Rhizaria: Cercozoa: Endomyxa-Phytomyxea: Phagomyxida: Phagomyxidae: Phagomyxa: Phagomyxa_sp. | 1.327 | 25.28 |
| OTU_19 | Alveolata: Ciliophora: Spirotrichea: Choreotrichida: Strombidinopsidae: Strombidinopsis: *Strombidinopsis acuminata* | 1.311 | 26.59 |
| OTU_36 | Alveolata: Ciliophora | 1.271 | 27.86 |
| OTU_24 | Alveolata: Dinoflagellata: Syndiniales: Dino-Group-I: Dino-Group-I-Clade-4: Dino-Group-I-Clade-4_X: Dino-Group-I-Clade-4_X_sp. | 1.189 | 29.05 |
| OTU_39 | Alveolata: Ciliophora: Spirotrichea: Choreotrichida: Strombidinopsidae: *Parastrombidinopsis* | 1.146 | 30.2 |
| OTU_18 | Alveolata: Dinoflagellata: Syndiniales: Dino-Group-I: Dino-Group-I-Clade-1: Dino-Group-I-Clade-1_X: Dino-Group-I-Clade-1_X_sp. | 1.115 | 31.31 |
| OTU_83 | Alveolata: Dinoflagellata: Dinophyceae | 1.089 | 32.4 |
| OTU_14 | Alveolata: Ciliophora: Spirotrichea: Strombidiida: Strombidiidae_N: *Strombidium*_N | 1.038 | 33.44 |
| OTU_38 | Alveolata: Ciliophora: Spirotrichea: Strombidiida | 1.027 | 34.46 |
