# Supplementary material for: Genetic Diversity, Community Assembly, and Shaping Factors of Benthic Microbial Eukaryotes in Dongshan Bay, Southeast China
Source: Front Microbiol. 2020 Dec 23;11:592489. doi: 10.3389/fmicb.2020.592489 (PMC7785585; doi:10.3389/fmicb.2020.592489)
Supplement: Supplementary file 1 [file Data_Sheet_1.zip › Table_S4.docx]

**TABLE S4** Contribution of various taxonomic groups to the dissimilarity of benthic microbial eukaryotic communities among the four groups identified in Fig. 4.

| Group | No. of OTUs | Contribution% |
| --- | --- | --- |
| Alveolate, Apicomplexa | 240 | 3.19 |
| Alveolate, Spirotrichea | 177 | 16.89 |
| Alveolate, Dinophyceae | 225 | 21.92 |
| Alveolate, Dino-Group-I | 116 | 4.70 |
| Stramenopiles, Bacillariophyta | 259 | 25.54 |
| Stramenopiles, Labyrinthulea | 77 | 0.450 |
| Stramenopiles, MAST | 68 | 0.50 |
| Stramenopiles, MOCH | 8 | 0.03 |
| Rhizaria, Filosa-Thecofilosea | 157 | 4.00 |
| Rhizaria, Filosa-Imbricatea | 174 | 2.20 |
| Rhizaria, Filosa-Granofilosea | 71 | 0.20 |
| Rhizaria, Endomyxa-Phytomyxea | 21 | 0.72 |
| Rhizaria, Endomyxa | 189 | 1.21 |
| Rhizaria, Radiolaria | 37 | 0.12 |
| Opisthokonta | 208 | 2.11 |
| Hacrobia | 108 | 0.78 |
| Excavata | 4 | <0.001 |
| Archaeplastida | 91 | 2.34 |
| Apusozoa | 54 | 0.16 |
| Amoebozoa | 83 | 0.26 |
