# Supplementary material for: Genetic Diversity, Community Assembly, and Shaping Factors of Benthic Microbial Eukaryotes in Dongshan Bay, Southeast China
Source: Front Microbiol. 2020 Dec 23;11:592489. doi: 10.3389/fmicb.2020.592489 (PMC7785585; doi:10.3389/fmicb.2020.592489)
Supplement: Supplementary file 1 [file Data_Sheet_1.zip › Table_S5.docx]

|  | Nodes | Edge | Degree | Positive edge (%) | Negative edge (%) | Network diameter | Density | Modularity | Average  clustering coefficient | Average  path length |
| --- | --- | --- | --- | --- | --- | --- | --- | --- | --- | --- |
| Nearshore | 449 | 2759 | 12.29 | 62.02% | 37.98% | 8 | 0.027 | 0.452 | 0.262 | 3.236 |
| Offshore | 500 | 6483 | 25.932 | 58.04% | 41.96% | 10 | 0.052 | 0.334 | 0.366 | 2.883 |

**TABLE S5.** Topological features of the benthic microbial eukaryotic networks of the Dongshan Bay.
