# Supplementary material for: Genetic Diversity, Community Assembly, and Shaping Factors of Benthic Microbial Eukaryotes in Dongshan Bay, Southeast China
Source: Front Microbiol. 2020 Dec 23;11:592489. doi: 10.3389/fmicb.2020.592489 (PMC7785585; doi:10.3389/fmicb.2020.592489)
Supplement: Supplementary file 1 [file Data_Sheet_1.zip › Table_S6.docx]

|  |  | Nearshore | | Offshore | |
| --- | --- | --- | --- | --- | --- |
| Groups | | Positive correlation (%) | Negative correlation (%) | Positive correlation (%) | Negative correlation (%) |
| Ciliophora | Bacillariophyta | 43.84 | 56.16 | 33.16 | 66.84 |
| Ciliophora | Dinophyceae | 62.65 | 37.35 | 62.60 | 37.40 |
| Dinophyceae | Bacillariophyta | 42.86 | 57.14 | 28.40 | 71.60 |
| Cercozoa | Bacillariophyta | **55.89** | **44.11** | **23.87** | **76.13** |
| Cercozoa | Ciliophora | 63.37 | 36.63 | 54.50 | 45.50 |
| Cercozoa | Dinophyceae | **48.86** | **51.14** | **76.54** | **23.46** |
| Syndiniales | Bacillariophyta | 56.67 | 43.33 | 69.23 | 30.77 |
| Syndiniales | Ciliophora | 40.91 | 59.09 | 40.48 | 59.52 |
| Syndiniales | Cercozoa | 55.17 | 44.83 | 50.00 | 50.00 |
| Syndiniales | Dinophyceae | **72.73** | **27.27** | **34.85** | **65.15** |

**TABLE S6.** The correlations between major microbial eukaryotic groups in nearshore and offshore sediments.
